# Evaluation of Electrochemical Process Improvement Using the Computer-Aided Nonlinear Frequency Response Method: Oxygen Reduction Reaction in Alkaline Media

**DOI:** 10.3389/fchem.2020.579869

**Published:** 2020-11-06

**Authors:** Luka A. Živković, Saikrishnan Kandaswamy, Menka Petkovska, Tanja Vidaković-Koch

**Affiliations:** ^1^Max Planck Institute for Dynamic of Complex Technical Systems, Magdeburg, Germany; ^2^Faculty of Technology and Metallurgy, University of Belgrade, Belgrade, Serbia

**Keywords:** forced periodic operation, silver rotating disc electrode, faradaic rectification, process intensification, cost–benefit indicator analysis, DC nonperiodic component, cNFR software, frequency domain

## Abstract

The intensification of an electrochemical process by forced periodic operation was studied for the first time using the computer-aided Nonlinear Frequency Response method. This method enabled the automatic generation of frequency response functions and the DC components (Faradaic rectification) of the cost (overpotential) and benefit (current density) indicators. The case study, oxygen reduction reaction, was investigated both experimentally and theoretically. The results of the cost–benefit indicator analysis show that forced periodic change of electrode potential can be superior when compared to the steady-state regime for specific operational parameters. When the electrode rotation rate is changed periodically, the process will always deteriorate as the dynamic operation will inevitably lead to the thickening of the diffusion layer. This phenomenon is explained both from a mathematical and a physical point of view.

## Highlights

- The DC component (Faradaic rectification) enables evaluation of the process intensification under the forced periodic regime in comparison to the steady-state regime.
- A routine for experimental determination of the DC component is given.
- The software for computer-aided Nonlinear Frequency Response method generates automatically all DC components of interest for the theoretical analysis.
- The cost–benefit indicator analysis was conducted by looking at the change of overpotential (cost) and current density (benefit indicator).
- The forced periodic change of electrode potential can intensify the oxygen reduction reaction process.
- The forced periodic change of just the electrode rotation rate will always deteriorate the oxygen reduction reaction process.

## Introduction

Understanding of the dynamics of electrochemical processes is of great interest for different emerging applications in the context of intermediate energy storage of fluctuating renewable energies and electromobility. Furthermore, dynamic methods are widely used for the diagnosis of electrochemical devices such as fuel cells (Wasterlain et al., 2010) and batteries (Moss et al., 2008), and kinetic mechanism discriminations (Krewer et al., 2006; Bensmann et al., 2010). In this respect, linear dynamic methods like electrochemical impedance spectroscopy (EIS) have found broad application in investigations of different electrochemical systems. However, due to linearity constraints, they show low discriminative power toward transient processes with similar time constants (Krewer et al., 2006). EIS is based on a frequency response of the investigated electrochemical system obtained for input modulations with small amplitudes, which should ensure its linear behavior.

The Nonlinear Frequency Response (NFR) method, which is obtained for larger input amplitudes, can provide more information for discrimination (Bensmann et al., 2010). The NFR of a weakly non-linear system is expressed as:

(1) $$\begin{array}{l} y_{qs}\left(t\right)=y_{s}+DC+h_{I}\left(t\right)+h_{II}\left(t\right)+h_{III}\left(t\right)+\ldots \end{array}$$

where *y*_*qs*_ is the system output (current or cell potential) after a periodic quasi-steady-state has been established, *y*_*s*_ is the output value in a steady-state operation, *DC* is the nonperiodic term of the NFR, and *h*_*i*_ (*i* = 1 – ∞) are the first- and higher-order harmonics of the NFR. Equation 1 is obtained when the system input (cell potential or current) is perturbed cosinusoidally around an established steady state. In the case of a linear system (small input amplitude), the DC component and higher-order harmonics in Equation 1 are equal to zero (Živković et al., 2020).

In electrochemical literature, the DC component was for the first time mentioned in the 1950s (Oldham, 1957) under the name Faradaic rectification (FR). In first applications, high-amplitude alternating current (A.C.) input signal was applied, and FR was referred to as a deviation of the average electrode D.C. potential from the steady-state potential value (Equation 1). The magnitude of the D.C. potential was found to be proportional to the square of the applied A.C. current and to be dependent also upon the rate constant and symmetry factor of the electrode reaction (Oldham, 1957). Therefore, the FR method was initially mainly considered for the determination of rate constants of fast electrochemical reactions.

Although the importance of nonlinear methods and FR was already at that time recognized, FR was for decades almost forgotten. In the late 1970s, the first applications related to corrosion research were reported. These studies were motivated by severe corrosion problems of underground power cables and a possible effect of A.C. on metal corrosion. Bertocci (1979) determined the amplitude and phase characteristics of harmonic components as well as FR and discussed their use for the determination of kinetic parameters of an electrode. He also concluded that under some conditions, A.C. would increase metal corrosion, but a proper choice of potential could minimize the effect. The method was revisited by Baranski and Diakowski (2006), who mainly concentrated on FR of the high-frequency region. They used high amplitude alternating potential as an input signal and observed that the average current value under forced periodic conditions deviated from its steady-state value. Baranski and Diakowski (2006) stated that it is not clear why researchers lost interest in the development and applications of FR techniques and assumed that the method was perhaps introduced too early. They conveyed that although FR measurements are easy to carry out, results are difficult to interpret, and data processing is very laborious. However, the authors expressed a strong belief that FR technique can be precious in modern electrochemical research, especially with respect to measurements of fast electrode kinetics.

All previous studies mainly concentrated on the determination of kinetic parameters of electrochemical reactions. These studies discussed FR theoretical backgrounds, but this was restricted to simple cases. Additionally, FR was treated (both in experiments and in theoretical derivations) separately from other parts of the nonlinear response (first- and higher-order harmonics). Furthermore, although FR is a nonperiodic term, it is dependent on frequency; therefore, it will have different values at different frequencies. This can be used 2-fold, (i) for discrimination of kinetic mechanisms based on the shape difference for different mechanisms, and (ii) for process intensification (Živković et al., 2020). Both aspects have not been significantly discussed in electrochemical literature. Concerning process intensification, the FR would indicate if the electrochemical process can be intensified or deteriorated by periodic operation (e.g., in terms of energy consumption and selectivity) when compared to the traditional steady-state regime. This aspect is of significant interest in the operation of electrochemical reactors under dynamic conditions.

In the present publication, the DC component (FR) is discussed in the context of NFR analysis, and experimental validation on an example of oxygen reduction reaction (ORR) under alkaline conditions is shown. The approach that we apply is based on the concept of higher-order frequency response functions (FRFs) (Bensmann et al., 2010). This concept was initially introduced through the NFR method in chemical engineering on examples like adsorption and chemical reaction systems (Petkovska, 2001; Petkovska and Seidel-Morgenstern, 2013). In the last decade, the NFR method was further developed on electrochemical examples like direct methanol fuel cell anode oxidation kinetics (Bensmann et al., 2010), ferrocyanide oxidation (Panić et al., 2011; Vidaković-Koch et al., 2011), and, recently, ORR kinetics (Kandaswamy et al., 2019). In these previous publications, the focus was on the analysis of nonlinearities contained in the higher-order harmonics, especially the second-order harmonic. Although we already introduced the DC component in these previous publications, we have not discussed it further.

To understand the DC component in the context of the NFR method, we recall shortly the general framework of this method (Marković et al., 2008). The NFR method is based on the Volterra series and the Fourier transform. For a cosinusoidal input modulation, the output can be expressed in the form of a Volterra series:

(2) $$\begin{array}{l} y_{qs}\left(t\right)=y_{s}+y_{1}\left(t\right)+y_{2}\left(t\right)+y_{3}\left(t\right)+\ldots \end{array}$$

where

(3) $$\begin{array}{l} y_{1}\left(t\right)=\left(\frac{A}{2}\right)\cdot e^{j\cdot \omega \cdot t}\cdot G^{\left(1\right)}\left(\omega \right)+\left(\frac{A}{2}\right){\cdot e}^{-j\cdot \omega \cdot t}\cdot G^{\left(1\right)}\left(-\omega \right) \end{array}$$

(4) $$\begin{array}{l} y_{2}\left(t\right)={\left(\frac{A}{2}\right)}^{2}\cdot e^{2\cdot j\cdot \omega \cdot t}\cdot G^{\left(2\right)}\left(\omega ,\omega \right)+2\cdot {\left(\frac{A}{2}\right)}^{2}{\cdot e}^{0}\cdot G^{\left(2\right)}\left(\omega ,-\omega \right)\\ \;+{\left(\frac{A}{2}\right)}^{2}{\cdot e}^{-2\cdot j\cdot \omega \cdot t}\cdot G^{\left(2\right)}\left(-\omega ,-\omega \right) \end{array}$$

etc., with *G*^(1)^( ω) being the first-order FRF (EIS admittance in case of potential input), and *G*^(2)^ (ω, ω) and *G*^(2)^ (ω, −ω) being the second-order symmetrical and asymmetrical FRF, respectively. *A* is the relative amplitude of the input change (with values from 0 to 1) around input's steady-state value, with forcing frequency ω. The FRFs, shown in Equations 3 and 4, are not input dependent and can be defined as dimensionless (present analysis) or have units of *A*·*m*^−2^·*V*^−*n*^, where *n* is the order of the FRF (1 for the first-order FRF, 2 for the second-order FRF, etc.). The FRFs are related to the harmonics of the output defined in Equation 1, according to:

(5) $$\begin{array}{l} DC=2\cdot {\left(\frac{A}{2}\right)}^{2}\cdot G^{\left(2\right)}\left(\omega ,-\omega \right)+6\cdot {\left(\frac{A}{2}\right)}^{4}\cdot G^{\left(4\right)}\left(\omega ,\omega ,-\omega ,-\omega \right)+\ldots \end{array}$$

(6) $$\begin{array}{l} h_{I}\left(t\right)=\left\{\left(\frac{A}{2}\right)\cdot G^{\left(1\right)}\left(\omega \right)+3\cdot {\left(\frac{A}{2}\right)}^{3}\cdot G^{\left(3\right)}\left(\omega ,\omega ,-\omega \right)+\ldots \right\}\cdot e^{\omega \cdot t\cdot j}\\ \;+\left\{\left(\frac{A}{2}\right)\cdot G^{\left(1\right)}\left(-\omega \right)+3\cdot {\left(\frac{A}{2}\right)}^{3}\cdot G^{\left(3\right)}\left(\omega ,-\omega ,-\omega \right)+\ldots \right\}\cdot e^{-\omega \cdot t\cdot j} \end{array}$$

(7) $$\begin{array}{l} h_{II}(t)=\left\{{\left(\frac{A}{2}\right)}^{2}\cdot G^{\left(2\right)}\left(\omega ,\omega \right)+4\cdot {\left(\frac{A}{2}\right)}^{4}\cdot G^{\left(4\right)}\left(\omega ,\omega ,\omega ,-\omega \right)+\ldots \right\}\cdot e^{2\omega \cdot t\cdot \;j}\\ \;+\{{\left(\frac{A}{2}\right)}^{2}\cdot G^{\left(2\right)}(-\omega ,-\omega )\\ \;+4\cdot {\left(\frac{A}{2}\right)}^{4}\cdot G^{\left(4\right)}(\omega ,-\omega ,-\omega ,-\omega )+\ldots \}\cdot e^{-2\omega \cdot t\cdot j} \end{array}$$

The experimental FRFs can be estimated from Equations 5–7, which was shown in previous publications (Panić et al., 2011; Kandaswamy et al., 2019) on the examples of the first- and second-order FRFs. As can be seen, the harmonics are influenced not only by the “main” FRF at the corresponding frequency but also by higher-order FRFs. For example, the first harmonic (Equation 6) contains contributions of *G*^(3)^(ω, ω, −ω) and the DC component (Equation 5) is influenced not only by the second-order asymmetrical FRF but also by higher-order asymmetrical FRFs. This aspect is of great importance for the experimental determination of FRFs. As we have shown previously (Panić et al., 2011; Kandaswamy et al., 2019), the amplitude of the input signal in an experiment has to be selected carefully to avoid contributions of the higher-order FRFs (similar holds for EIS, but with restriction to the linear part of the signal).

The FRFs that are shown in Equations 3–7 form the dynamic model of the investigated nonlinear system and are inherently related to its mechanism and parameters. In many cases, the shape of the second-order FRF enables mechanism discrimination, as was already demonstrated on an example of methanol oxidation kinetics by Bensmann et al. (2010). In general, this should also hold for the second-order asymmetrical function *G*^(2)^(ω, −ω), which is the main part of the DC component (FR). Additionally, this asymmetrical function shows if the process can be intensified or deteriorated by periodic operation, which is demonstrated further, by analyzing the second-order asymmetrical function *G*^(2)^(ω, −ω) for two different inputs (electrode potential and rotation rate). As already pointed out by Baranski and Diakowski (2006), the DC results are difficult to interpret without a theoretical background. Therefore, the experimental analysis has to be guided by theory.

The analytical expressions of the second-order asymmetrical FRF (the main part of DC component), as well as other FRFs, can be derived starting from a non-linear dynamic model of the investigated system based on the procedure that is well-established and documented in several publications (Petkovska, 2005; Petkovska and Seidel-Morgenstern, 2013). However, the derivation of these analytical expressions requires both time and specific mathematical skills from the user, thus making its application unappealing to beginners, especially for very complex mechanisms. Therefore, a software tool for automatic derivation of theoretical FRFs, a so-called computer-aided Nonlinear Frequency Response (cNFR) method, was developed by Živković et al. (2020). The feasibility of the cNFR method was already demonstrated in our previous publication (Kandaswamy et al., 2019), and this method will be discussed in more detail in the forthcoming publication (Živković et al., 2020). The cNFR method is a model-based tool that can be used on any stable system that can be mathematically described by equations with differentiable and continuous terms. The software for the cNFR method enables fast and automatic derivation of all analytical FRFs of interest, through a user-friendly modeling interface. Most importantly, cNFR-generated FRF files allow for smooth integration with existing numerical algorithms, the result of which can be fast parameter estimation for the competing reaction mechanisms, and optimization of the operating variables. In the present publication, this tool was used for the derivation of the second-order asymmetrical FRFs for two different inputs.

## Determination of the Theoretical Second-Order Asymmetrical FRF for ORR Using the cNFR Method

The ORR under alkaline conditions is of high interest in many existing and emerging applications, e.g., in chloralkali electrolysis with oxygen-depolarized cathode or in metal/air batteries. Among different catalysts, silver seems to be quite promising material, catalyzing 4 e- at low overpotentials. In our previous publication (Kandaswamy et al., 2019), we studied ORR on silver under alkaline conditions using cNFR analysis. We have shown that a simple nonlinear kinetic model of ORR can describe ORR kinetics in low concentrated NaOH solution (0.1 M) very well. Shortly, the model includes a mass balance equation for the concentration of oxygen, *c*:

(8) $$\begin{array}{l} \frac{\partial c\left(z,t\right)}{\partial t}=D\cdot \frac{\partial^{2}c\left(z,t\right)}{\partial z^{2}} \end{array}$$

where *D* is the diffusion coefficient of oxygen in the NaOH solution. The boundary conditions are determined by the ORR reaction rate, *r*, at the electrode interface and oxygen concentration in the bulk, *c*_*bulk*_:

(9) $$\begin{array}{l} D\cdot {\;\frac{\partial c}{\partial z}|}_{z=0}=r(t) \end{array}$$

(10) $$\begin{array}{l} c\left(z=\delta ,t\right)=\;c_{bulk} \end{array}$$

For the rotating disc electrode, diffusion layer thickness, δ, depends on the electrode rotation rate, ω_*r*_, according to the well-known dependence:

(11) $$\begin{array}{l} \delta \left(t\right)=1.61\cdot D^{\frac{1}{3}}\cdot \nu^{\frac{1}{6}}\cdot {\omega_{r}\left(t\right)}^{-\frac{1}{2}} \end{array}$$

where ν is the kinematic viscosity of the liquid solution.

The ORR model also contains the equation for the charge balance (Kandaswamy et al., 2019):

(12) $$\begin{array}{l} c_{dl}\cdot \frac{d\eta \left(t\right)}{dt}=curr\left(t\right)+F\cdot 4\cdot r\left(t\right) \end{array}$$

where *c*_*dl*_ is the double-layer capacitance, η is the electrode overpotential, *F* is Faraday's constant, and *curr* is current density, which is also the main output of interest. The overpotential is further defined as (Kandaswamy et al., 2019):

(13) $$\begin{array}{l} \eta \left(t\right)=E\left(t\right)-E^{\theta }-R_{\Omega }\cdot curr\left(t\right) \end{array}$$

where *E* and *E*^θ^ are the electrode and the reversible electrode potential, respectively, and *R*_Ω_ is ohmic resistance.

The kinetics of the ORR is described with the help of Tafel equation assuming first electron transfer as a rate-determining step:

(14) $$\begin{array}{l} r\left(t\right)=k_{e1}\cdot e^{-\frac{\alpha \cdot F}{R\cdot T}\cdot \eta \left(t\right)}\cdot c\left(0,t\right) \end{array}$$

where *k*_*e*1_ is the ORR kinetic constant, α is transfer coefficient, *R* is the universal gas constant, and *T* is the reaction temperature.

For the automatic derivation of the necessary FRFs, the mass balance equation (Equation 8) was discretized (please see Supporting Information) and the cNFR method was used by running the application described in detail by Živković et al. (2020). The FRFs can be derived for different combinations of the outputs (e.g., the overpotential, current density, reaction rate, diffusion layer thickness) and the inputs, or periodically changed variables (e.g., electrode potential and the rotation rate). For the analysis in this publication, primary outputs of interest were the current density, *curr*, as the benefit indicator in our process improvement and overpotential, η, as the cost indicator. The overpotential was considered as a most representative cost indication for the sole ORR process. If the investigation, and thus mathematical model, was extended on the reactor level, overall power would be a good cost indication. Our choice of cost and benefit indicators means that process will be considered intensified when the absolute value of the benefit indicator, i.e., *curr*, increases, and the absolute value of the cost indicator, i.e., η, decreases.

Two different inputs were considered, the electrode potential and rotation rate:

(15) $$\begin{array}{l} E\left(t\right)=E_{s}\cdot \left[1+A_{1}\cdot cos\left(\omega \cdot t\right)\right] \end{array}$$

(16) $$\begin{array}{l} \omega_{r}\left(t\right)=\omega_{r,s}\cdot \left[1+A_{2}\cdot cos\left(\omega \cdot t\right)\right] \end{array}$$

where *E*_*s*_ and ω_*r,s*_ are the steady-state values of electrode potential and rotation rate, respectively, around which the forced periodic operation was performed with the frequency, ω, and the relative amplitude of change, *A*_1_ and *A*_2_.

In electrochemistry, electrical input variables (potential or current) are commonly considered. Additionally, nonelectrical inputs could be of interest, e.g., the concentration in the bulk or temperature, as they directly influence the reaction rate (Equation 14). The dynamic methods using nonelectrical inputs are also discussed in electrochemistry, but the theoretical analysis was restricted so far to the linear range as shown by Sorrentino et al. (2017) and others (Tokuda et al., 1975; Tribollet and Newman, 1983; Olivier et al., 1992). The cNFR method enables the full treatment of both electrical and nonelectrical inputs in the linear as well as nonlinear range, as it will be shown here on the example of the forced periodic change of electrode rotation rate and electrode potential. The periodic change of the electrode potential was covered in part by Kandaswamy et al. (2019). The electrode rotation rate is a good choice for RDE, since it is electrically modulated, and it directly influences oxygen concentration at the electrode surface through the thickness of the diffusion layer. It should be mentioned that in the linear range, the electrode rotation rate as an input was already introduced by Tokuda et al. (1975) and Tribollet and Newman (1983). The method was termed electro-hydro-dynamical impedance (Tribollet and Newman, 1983).

## Experimental Derivation of FRFs

**Figure 1:**
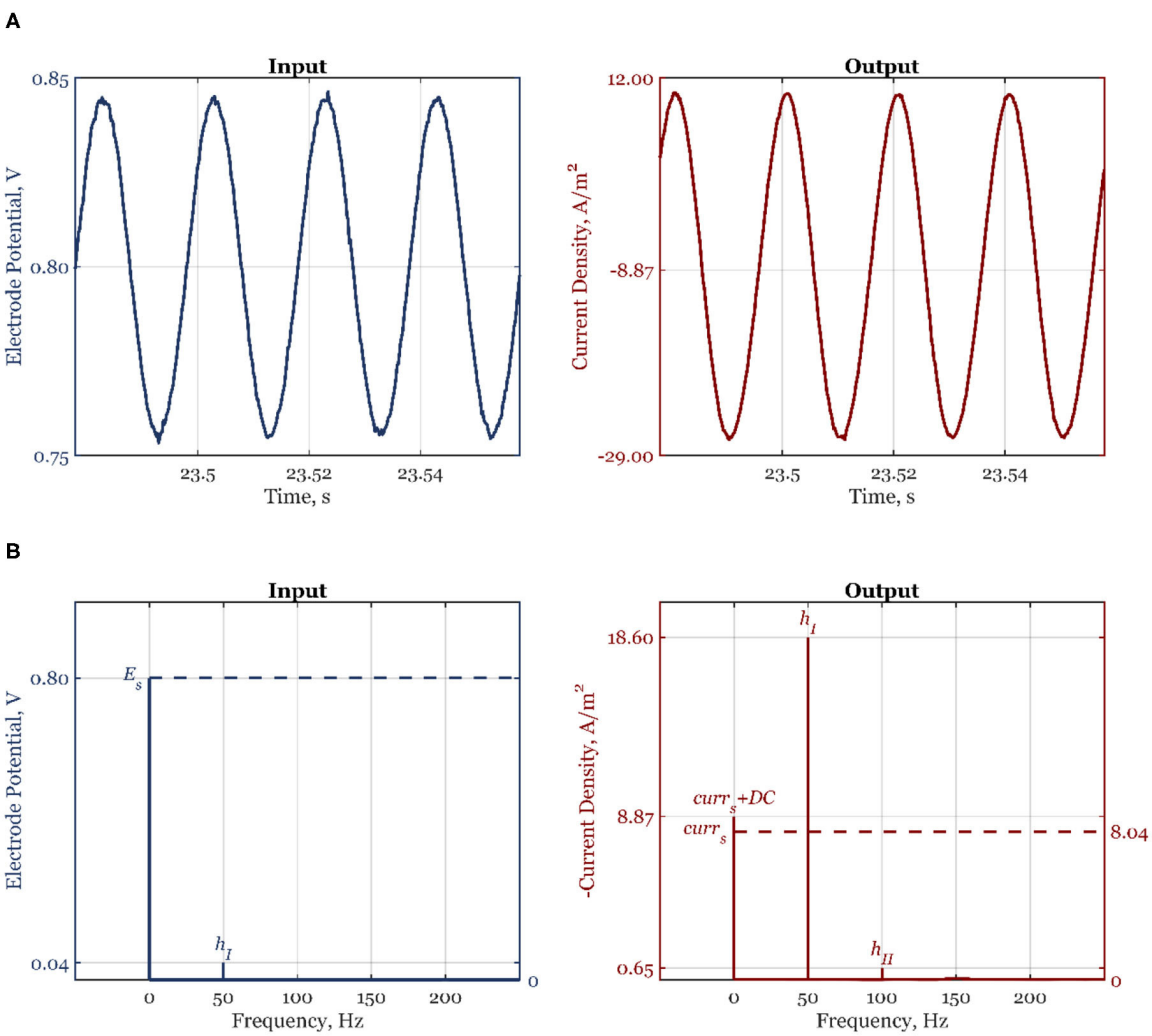
Experimental time-domain **(A)** and frequency-domain **(B)** signals of the electrode potential, and current density, for the forcing frequency *f* = 50 Hz, amplitude *A*_1_ = 0.053, and the steady-state electrode potential value *E*_*s*_ = 0.8 V.

All experiments were performed in a custom-made three-compartment Teflon half-cell with working electrode (WE) and counter electrode (CE) compartments separated by Nafion membrane and reference electrode connected to the WE compartment via a Luggin capillary. Both compartments were filled with 0.1 M NaOH electrolyte (sodium hydroxide monohydrate 99.99% Suprapur® Merck - B1491866739). The electrolyte solution was saturated with either argon (Linde plc, purity grade 5.0) or oxygen (Linde plc, purity grade 5.0) gasses without further purification. Polycrystalline sliver rotating disk electrode (E5TPK -AFE5T050XXPK, Pine Research Instrumentation, Inc.) was the WE, platinum wire (AFCTR5, Pine Research Instrumentation, Inc.) was the CE, and reversible hydrogen electrode (HydroFlex electrode from Gaskatel GmbH, Germany) was the reference electrode. The WE was connected to a modulated speed rotator (AFMSRCE, Pine Research Instrumentation, Inc.). Before measurement, WE was polished by 0.3 μm alumina suspension (AK POLISH Pine Research) followed by 2 min sonication in an ultrasonic bath (Sonorex RK31, Bandelin electronic GmbH & Co. KG). This procedure was repeated by polishing with 0.05-μm alumina suspension (AK POLISH Pine Research) and 2-min sonication. Before further experiments, the WE was electrochemically preconditioned by potential cycling between 0 and 1 V (30 cycles at a scan rate 200 mV s^−1^) in argon saturated electrolyte. After that, the electrolyte was saturated with oxygen and WE was cycled in the potential range between 0 and 1 V (10 cycles at a scan rate 50 mV s^−1^). The NFR measurements were performed at 1,600 rpm and the potential value of 0.8 V vs. RHE. For these measurements, the electrode potential input amplitude was 5.3% of the steady-state value, i.e., 42.4, or 30 mV RMS. Experiments were performed at each frequency of interest separately with Solartron Analytical EnergyLab XM potentiostat and its software. The number of frequencies in the input was chosen to be 1 and the option measure nonstimulated harmonics in the output was selected. Between two measurements at a single frequency, electrochemical preconditioning, including potential cycling and a potentiostatic step, was always introduced. The analysis of signals was performed with the help of Fast Fourier Transform (FFT). The frequency value of zero (nonperiodic contribution) was assigned to the sum of the steady-state value of the output variable and their corresponding DC component. In Figure 1A, a raw signal of the input, electrode potential, and output, the current density, can be seen. In Figure 1B, corresponding FFT diagrams of the input and output are shown. As expected, the input has only the first harmonic, *h*_*I*_, at the fundamental frequency (*f* = 50 Hz) and the nonperiodic part, which corresponds to steady-state potential value, *E*_*s*_. The output shows two harmonics, *h*_*I*_ and *h*_*II*_, at the fundamental frequency (*f* = 50 Hz) and 2-fold of fundamental frequency, respectively (100 Hz). The higher-order harmonics are missing as the amplitude of input change was chosen so that their contributions are negligible (Figure 1B). The nonperiodic term corresponds to the sum of the current steady-state value and the DC component.

The values obtained by FFT are then used to calculate the experimental second-order asymmetrical FRF, which is the main part of the DC component according to the equation:

(17) $$\begin{array}{l} \;G_{E,curr}^{\left(2\right)}\left(\omega ,-\omega \right){|}_{\text{exp}}=\frac{DC_{E,curr}}{curr_{s}}\cdot 2\cdot {\left(\frac{E_{s}}{A_{d,1}}\right)}^{2} \end{array}$$

where *DC*_*E,curr*_ is the nonperiodic term in the output (without the steady-state value *curr*_*s*_) and *A*_*d*,1_ is the dimensional amplitude change (42.4 mV) of the first input signal, potential, around its steady-state, *E*_*s*_. As can be seen, the experimental $G_{E,curr}^{\left(2\right)}\left(\omega ,-\omega \right)$ is expressed in dimensionless form, as the theoretical FRF present in Equation 5.

In the present publication, only experimental validation of DC component determination for potential as an input has been demonstrated. The experimental validation of other FRFs derived with the cNFR method was shown by Kandaswamy et al. (2019). Here, for the first time, the cNFR method was used not for an experimental identification study but for a process intensification analysis of a forced periodic ORR process, which is an integral part of the ORR reactor.

## Results and Discussion

### Analysis of the Asymmetrical Second-Order FRF

**Figure 2:**
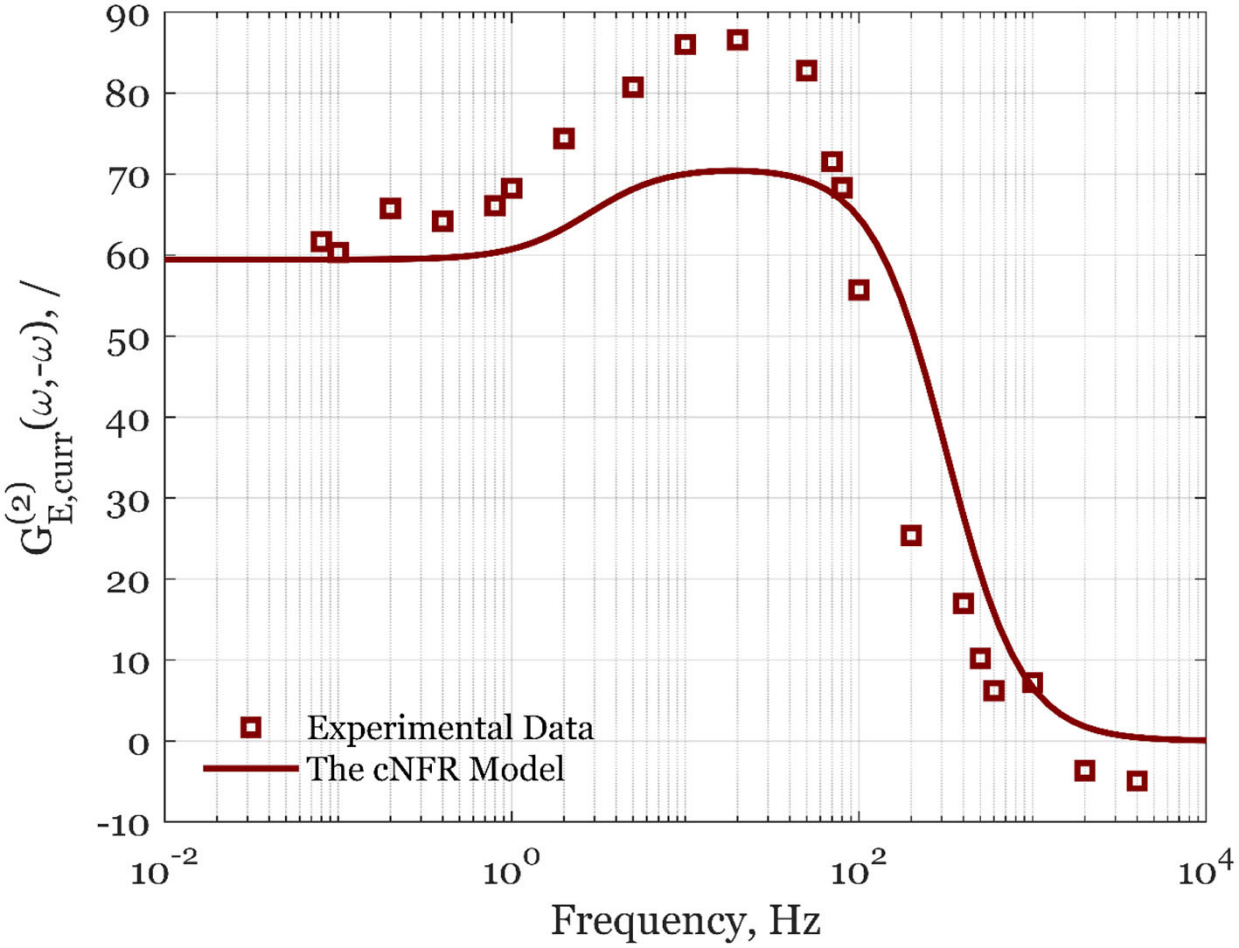
Averaged experimental data (squares) and simulated cNFR results (line) of the second-order asymmetrical FRF for the potential, *E*, as the input, and current density, *curr*, as the output at the steady-state potential of 0.8 V and 1,600 rpm.

**Figure 3:**
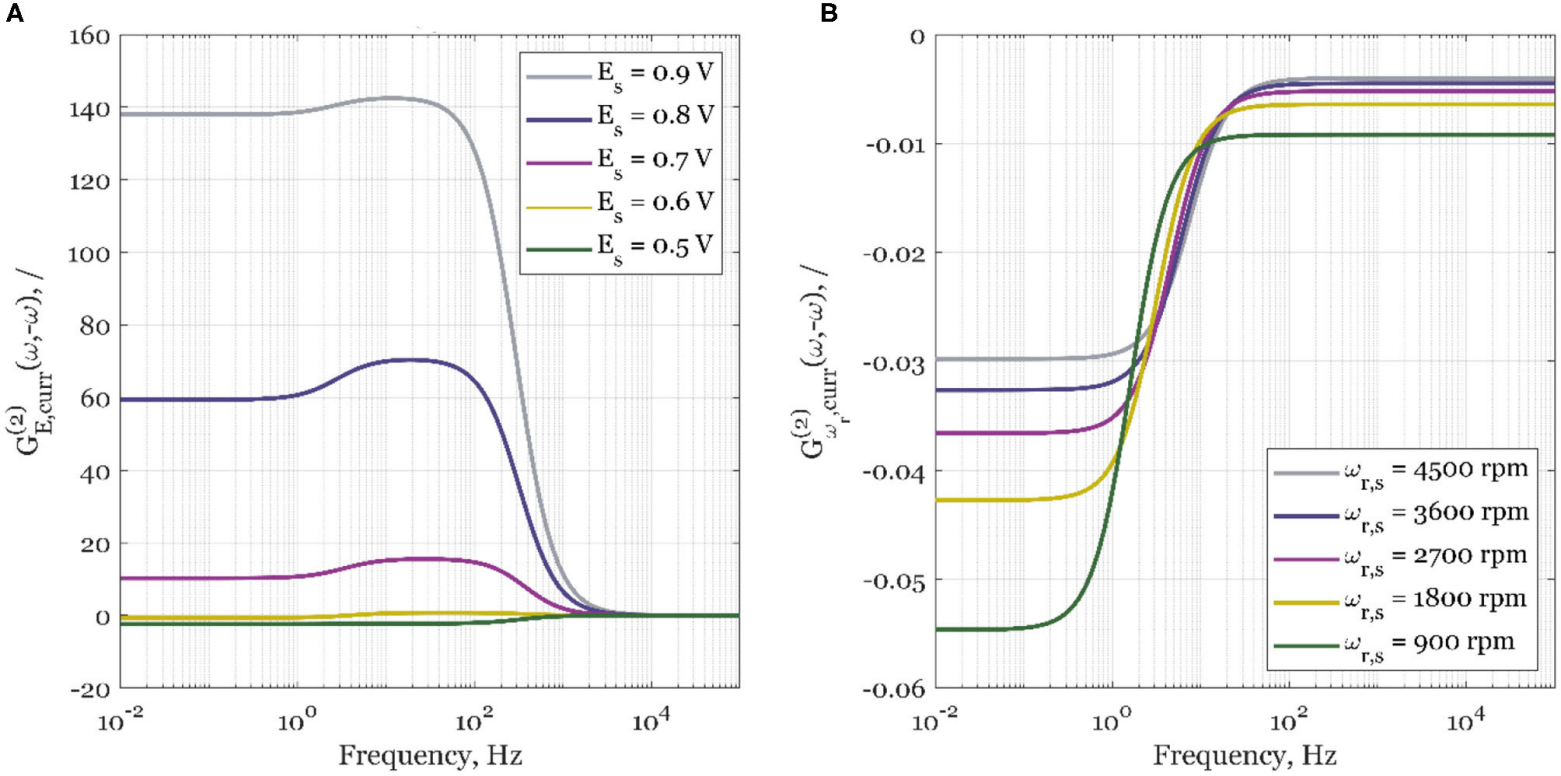
The simulated second-order asymmetrical FRFs for the current density, *curr*, as the output, and **(A)** electrode potential, *E*, as the periodically changed input, and steady-state rotation rate 1,600 rpm; **(B)** electrode rotation rate, ω_*r*_, as the periodically changed input, and steady-state potential 0.8 V.

The calculated values of asymmetrical second-order FRFs for forced periodic change of different potentials and rotation rates are shown in Figures 2, 3. In Figure 2, the experimental data are shown for the steady-state electrode rotation rate of 1,600 rpm, and a potential of 0.8 V. The asymmetrical FRF for potential input changes continuously from high to low frequencies (Figure 2). At high frequencies, there is no deviation between the steady-state current value and the average value under forced periodic operation; therefore, the second-order asymmetrical FRF is zero at high frequencies. With an increase of frequency, the second-order asymmetrical FRF is increasing, reaching a broad maximum in the frequency range between ca. 10 and 100 Hz. At low frequencies (below 1 Hz), a plateau can be observed. The maximum is observed in the frequency range that is dominated by reaction kinetics (Kandaswamy et al., 2019), while the plateau lies in the region limited by mass transfer. The simulated data used parameter values determined in our previous publication (Kandaswamy et al., 2019), and no attempt of data fitting was carried out (the parameter values are provided in SI). The experimental validation in Figure 2 shows a good qualitative agreement of the averaged experimental data sets with the simulation of the automatically generated cNFR model.

In Figure 3A, the influence of steady-state potential value on simulated second-order asymmetrical FRFs is shown. As shown by Kandaswamy et al. (2019), the selected steady-state potential values cover a broad range of potentials, where the overall control of ORR kinetics changes from almost pure kinetic control (0.9 V) to mass transport control (0.5 V). As can be seen, for the potential of 0.5 V, there will be no process improvement in the whole frequency range as the sign of the FRF is negative. As the steady-state electrode potentials increase, the periodic process improvement is expected to be higher (increased positive $G_{E,curr}^{\left(2\right)}\left(\omega ,-\omega \right)$ value) when compared to the steady-state operation. The process intensification will happen only if the forcing frequencies of electrode potential are below 1 kHz. The results show that the highest improvement is expected in the kinetically controlled region (0.9 V). At more negative potentials, the ORR is slowed down by mass transport. Therefore, the value of the second-order asymmetrical function is decreasing at more negative potentials, reaching negative values at 0.5 V (pure mass transport control).

Since we have seen that periodic change of electrode potential can especially intensify the electrochemical process under conditions of kinetic control, but it deteriorates the process under conditions of mass transfer control, we checked further how the change of mass transfer conditions induced by the periodic change of the electrode rotation rate influences the process.

Surprisingly, when electrode rotation rate, ω_*r*_, is forced to periodically change around its steady-state value, ω_*r, s*_, the second-order asymmetrical FRF gains a negative sign in the whole frequency range and at all studied rotation rate values. Consequently, the steady-state operation will be always superior to the forced periodic one (Figure 3B). This negative effect is more expressed at lower steady-state rotation rates (900 rpm), than at higher (4,500 rpm). The less expressed effect at higher steady-state rotation rates is expected since the overall kinetics at higher rotation rates is less influenced by mass transport.

From Equation 11, it can be seen that the rotation rate directly affects only the thickness of the diffusion layer. Therefore, to understand the effect of forced periodic change of rotation rate on ORR kinetics, it is instructive to investigate the second-order asymmetrical FRF for the diffusion layer thickness, δ. In this respect, Equation 11 is rewritten in a more general form as:

(18) $$\begin{array}{l} \delta \left(t\right)=1.61\cdot D^{\frac{1}{3}}\cdot \nu^{\frac{1}{6}}\cdot {\omega_{r}\left(t\right)}^{n} \end{array}$$

where *n* is the power of the rotation rate. The cNFR-derived second-order FRF for the diffusion layer thickness is then expressed as:

(19) $$\begin{array}{l} G_{\omega_{r},\delta }^{\left(2\right)}=\frac{n\cdot \left(n-1\right)}{2} \end{array}$$

From Equation 19, it follows that the second-order FRF for the diffusion layer thickness, δ, as the output, and electrode rotation rate, ω_*r*_, as the input, depends only on the power of the rotation rate, *n*. Therefore:

1. For *n* = 0 or *n* = 1: Equation 18 becomes linear as *D* and ν are constants. This results in $G_{\omega_{r},\delta }^{\left(2\right)}$ (Equation 19) being zero (no change in diffusion layer thickness when compared to the steady-state operation).
2. For 0 < *n* < 1: The sign of $G_{\omega_{r},\delta }^{\left(2\right)}$ will always be negative, meaning that the diffusion layer will be thinner in the forced periodic operation when compared to the steady-state one. The thinner layer would in return result in higher current densities and lead to process intensification.
3. For *n* < 0 or *n* > 1: The sign of $G_{\omega_{r},\delta }^{\left(2\right)}$ will always be positive, meaning that the diffusion layer will be thicker in the forced periodic operation when compared to the steady-state one. The thicker layer would in return give lower current densities and lead to process deterioration.

In the present case (Equation 11), *n* = −1/2, which corresponds to the third case scenario. As diffusion layer thickness is always inversely proportional (*n* < 0) to the electrode rotation rate (higher rates lead to thinner layers), $G_{\omega_{r},\delta }^{\left(2\right)}$ will always be positive, assuming constant values of the diffusion coefficient and the dynamic viscosity.

### Cost–Benefit Indicator Analysis Using the Corresponding DC Components

In the previous section, the dimensionless second-order asymmetrical FRFs were discussed. For the cost–benefit indicator analysis of the periodic regime, the mean value of the overpotential, as the cost indicator, and the mean value of the current density, as the benefit indicator, will be compared with the corresponding steady-state regime values. In the case when electrode potential, *E*, is periodically changed around its steady-state value, *E*_*s*_, the mean value of the benefit indicator in the periodic regime is:

(20) $$\begin{array}{l} \bar{curr}=curr_{s}\cdot \left(1+DC_{E,curr}\right)\approx curr_{s}\cdot \\ \;\left[1+2\cdot {\left(\frac{A_{1}}{2}\right)}^{2}\cdot G_{E,curr}^{\left(2\right)}\left(\omega ,-\omega \right)\right] \end{array}$$

while for electrode rotation rate, ω_*r*_, as the forced variable, it becomes:

(21) $$\begin{array}{l} \bar{curr}=curr_{s}\cdot \left(1+DC_{\omega_{r},curr}\right)\approx curr_{s}\cdot \\ \;\left[1+2\cdot {\left(\frac{A_{2}}{2}\right)}^{2}\cdot G_{\omega_{r},curr}^{\left(2\right)}\left(\omega ,-\omega \right)\right] \end{array}$$

Analogously, mean output values of the overpotential, $\bar{\eta }$, are calculated using the DC components for the overpotential as the output, *DC*_*E*, η_ and *DC*_ω_*r*_, η_:

(22) $$\begin{array}{l} \bar{\eta }=\eta_{s}\cdot \left(1+DC_{E,\eta }\right)\approx \eta_{s}\cdot \left[1+2\cdot {\left(\frac{A_{1}}{2}\right)}^{2}\cdot G_{E,\eta }^{\left(2\right)}\left(\omega ,-\omega \right)\right] \end{array}$$

while for electrode rotation rate, ω_*r*_, as the forced variable, it becomes:

(23) $$\begin{array}{l} \bar{\eta }=\eta_{s}\cdot \left(1+DC_{\omega_{r},\eta }\right)\approx \eta_{s}\cdot \left[1+2\cdot {\left(\frac{A_{2}}{2}\right)}^{2}\cdot G_{\omega_{r},\eta }^{\left(2\right)}\left(\omega ,-\omega \right)\right] \end{array}$$

**Figure 4:**
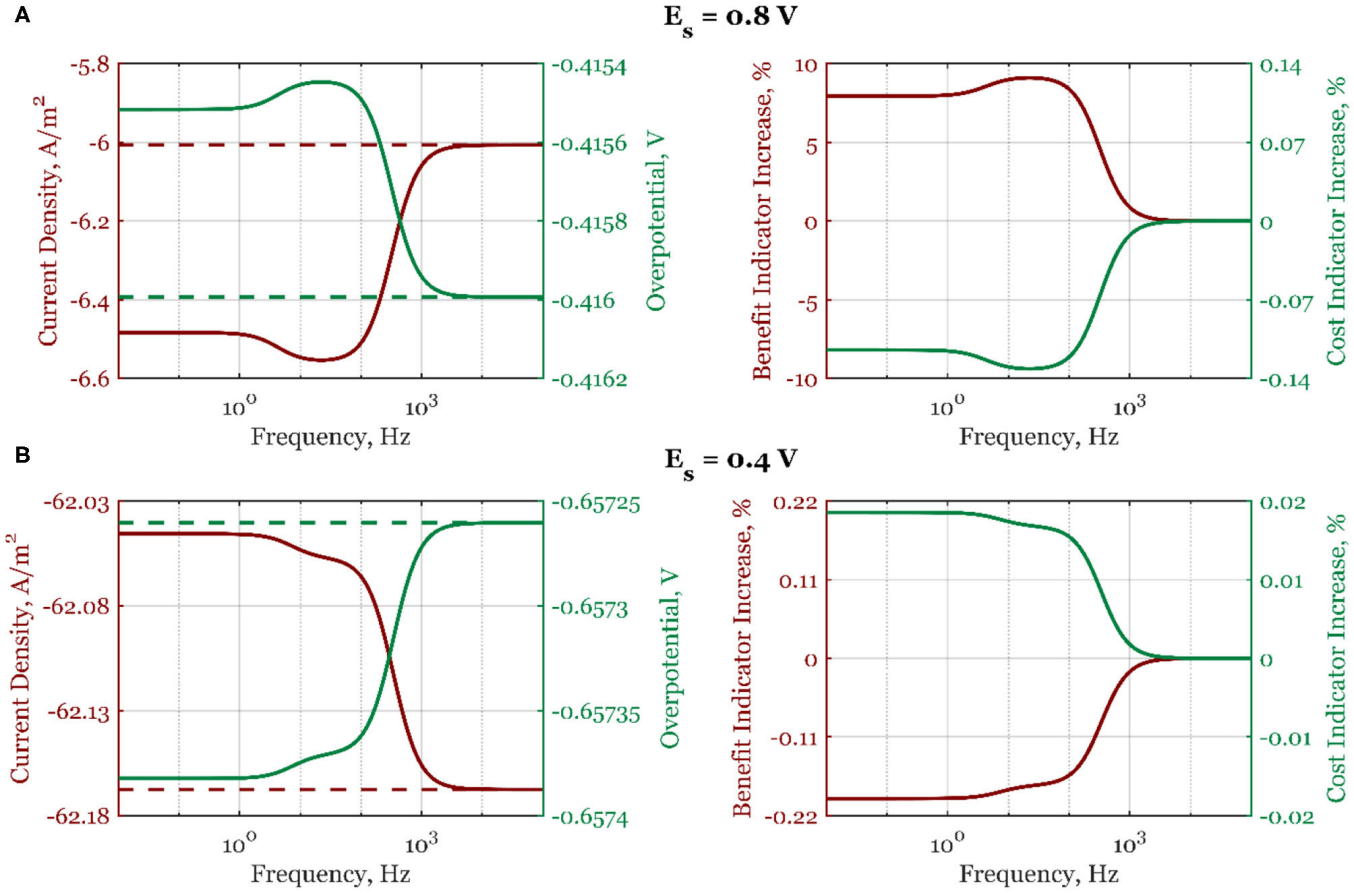
The mean current density and overpotential values (left side) for steady-state (dashed line) and dynamic regime (solid line), and the corresponding dynamic regime benefit and cost indicator increases relative to the steady-state regime (right side) when electrode potential is periodically changed with the amplitude of 5%, steady-state electrode rotation rate ω_*r,s*_ = 2,500 rpm, and electrode potentials: **(A)** *E*_*s*_ = 0.8 V and **(B)** *E*_*s*_ = 0.4 V, in the frequency range of 10^−2^-10^5^ Hz.

In Figure 4, the cost and benefit indicators are shown side by side when electrode potential is the forcing parameter. Data at two different steady-state potential values, 0.8 V (a) and 0.4 V (b), are represented. As can be seen, at *E*_*s*_ = 0.8 V, the absolute mean current density, $\bar{curr}$, shown with a solid line increases continuously from the high- to the low-frequency range (Figure 4A), following the form of the second-order asymmetrical FRF shown in Figure 2. At high frequencies, there is no process improvement when compared to the steady-state operation (dashed line). Likewise, the cost indicator (Figure 4A) also shows a decrease in the low-frequency range (the overpotential becomes more positive). At steady-state potential value in the region of dominant mass transfer control (*E*_*s*_ = 0.4 V, Figure 4B), the absolute mean current density value is lower than under steady-state conditions, thus deteriorating the electrochemical process. All process indicators are worsened compared to the steady-state operation (overpotential is more negative than in the steady state, the process benefit is decreasing, and the cost indicator is increasing).

Further simulation results (Supplementary Figure 1) at the same steady-state potential values (0.6 V) but different steady-state rotation rates (1,600 and 3,600 rpm) show that the process intensification is more expressed at higher rotation rates (maximum benefit indicator is changing from 1.9% at 1,600 rpm to 2.3% at 3,600 rpm). The higher rotation rates lead to thinner diffusion layers, which reduce the influence of the mass transport on the mean values of current densities and overpotentials.

**Figure 5:**
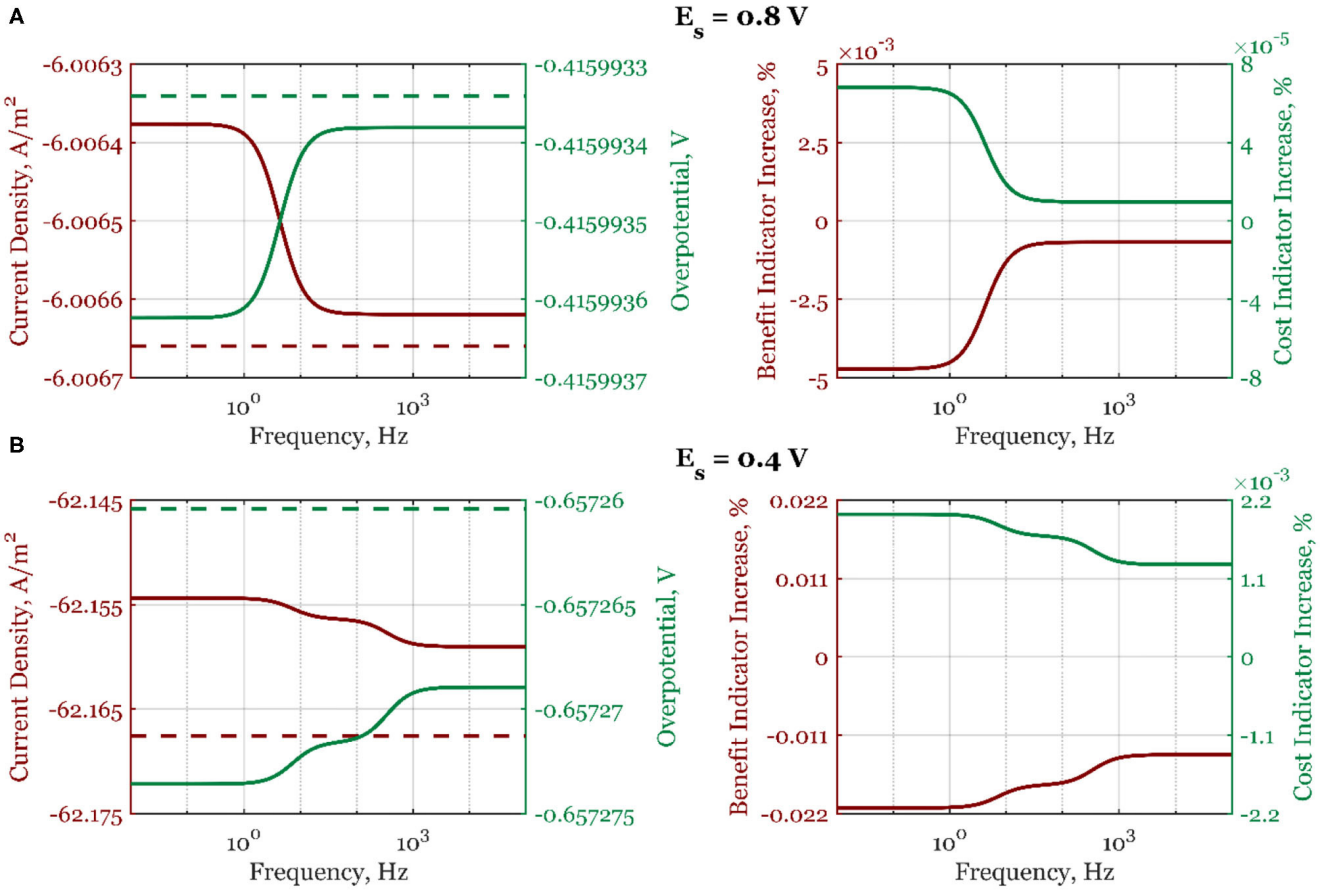
The mean current density and overpotential values (left side) for steady-state (dashed line) and dynamic regime (full line), and the corresponding dynamic regime benefit and cost indicator increases relative to the steady-state regime (right side) when electrode rotation rate is periodically changed with the amplitude of 5% around the value ω_*r, s*_ = 2,500 rpm, and for steady-state electrode potentials: **(A)** *E*_*s*_ = 0.8 V and **(B)** *E*_*s*_ = 0.4 V, in the frequency range of 10^−2^-10^5^ Hz.

A similar analysis is done when the electrode rotation rate is the forcing parameter (Figure 5, Supplementary Figure 2). In the whole frequency range, the process deterioration will happen if the forced periodic regime is used. As explained, this is due to the thickening of the diffusion layer when the electrode rotation rate is periodically changed (Equation 19).

However, as previously shown by Nikolić et al. (2015), when two inputs are simultaneously modulated with the correctly selected phase angle between them, the process intensification can be achieved even when the single-input change shows process deterioration. The multi-input periodic change analysis will be covered in our future publication.

## Conclusions

The focus in the present publication was to demonstrate a possibility of ORR process intensification by the forced periodic operation. Two different periodic inputs were considered (potential and electrode rotation rate). Analytical expressions of the second-order asymmetrical FRFs were derived with the help of cNFR software for automatic derivation of FRFs and DC components (also known as Faradaic rectification and related to the second-order asymmetrical FRF). As shown, second-order asymmetrical FRFs (DC components) give valuable information about system dynamics in electrochemistry. No expensive and time-consuming numerical integration is needed to evaluate mean output variable values, which can be used as benefit and cost indicators in the process improvement analysis. In this paper, only single-input forced periodic operations were considered, with several important conclusions:

1. *The ORR dynamic process will be intensified when compared to the steady-state regime if the electrode potential is forced to periodically change around its steady state under certain operating conditions*.

Process intensification will happen at frequencies below 1 kHz with the maximum improvement in the kinetically controlled region (between ca. 10 and 1 kHz). The cost indicator, overpotential, will also decrease in this region. At high frequencies, there will be no change in process improvement when compared to the steady-state regime. At lower potential steady-state values, the ORR dynamic process will deteriorate.

2. *The ORR dynamic process will inevitably deteriorate when compared to the steady-state regime if the electrode rotation rate is forced to periodically operate around its steady state*.

Process deterioration will happen in the whole frequency region as the dynamic change of the electrode rotation rate will always lead to thicker diffusion layers, thus giving lower current densities when compared to the steady-state regime.

3. *The cNFR method allowed for fast and easy analysis of different operational dynamic regimes with the automatic generation of FRFs and without the need to solve the systems of ordinary differential equations*.

In this paper, the DC components of a complex system were analyzed according to the well-established NFR method and the concept of higher-order FRFs. This was possible thanks to the automatic derivation of FRFs and generated Matlab codes by the cNFR software. The codes were then used for experimental identification and subsequent simulations and quick cost–benefit indicator preliminary analysis. Only the single-input periodic change was considered in this publication, although the cNFR method gives information about the simultaneous change for different selected input combinations. As will be shown in the next publication, the process intensification analysis used here can be further extended to rigorous multi-objective optimization, for finding the optimal parameters that give the highest benefit and lowest cost indicator values.

## Data Availability Statement

All datasets presented in this study are included in the article/Supplementary Material.

## Author Contributions

LŽ and TV-K: conceptualization and writing—original draft preparation. LŽ, MP, and TV-K: data analysis and writing—review and editing. SK: experimental investigation. TV-K and MP: supervision. All authors contributed to the article and approved the submitted version.

## Conflict of Interest

The authors declare that the research was conducted in the absence of any commercial or financial relationships that could be construed as a potential conflict of interest.
