# Supplementary material for: Evaluation of Electrochemical Process Improvement Using the Computer-Aided Nonlinear Frequency Response Method: Oxygen Reduction Reaction in Alkaline Media
Source: Front Chem. 2020 Nov 6;8:579869. doi: 10.3389/fchem.2020.579869 (PMC7677355; doi:10.3389/fchem.2020.579869)
Supplement: Supplementary file 1 [file Data_Sheet_1.pdf]

Supporting Material

Title:

Evaluation of electrochemical process improvement using computer-aided nonlinear frequency response method: Oxygen reduction reaction in alkaline media

Authors:

Luka A. Živković[a,*], Saikrishnan Kandaswamy[a], Menka Petkovska[b], Tanja Vidaković-Koch[a,*]

a – Max Planck Institute for Dynamic of Complex Technical Systems, Magdeburg, Germany

b – Faculty of Technology and Metallurgy, University of Belgrade, Serbia

* – Corresponding authors:

Luka A. Živković (zivkovic@mpi-magdeburg.mpg.de)

Tanja Vidaković-Koch (vidakovic@mpi-magdeburg.mpg.de)

A. Discretized form of the mass balance model equations

The discretized form of Eq. 8-10 is:

$$\frac{dc_1(t)}{dt} = \frac{D}{\delta(t)^2/2} \cdot \left(c_{bulk} - c_1(t)\right) - \frac{D}{\delta(t)^2/2} \cdot \frac{c_1(t) \cdot k_{app}(t)}{D/\delta(t) + k_{app}(t)} \qquad \text{A. 1}$$

where c_1 is the intermediary oxygen concentration, and k_{app} the apparent kinetic variable:

$$k_{app}(t) = k_{e1} \cdot e^{-\frac{\alpha \cdot F}{R \cdot T} \cdot \eta(t)} \qquad \text{A. 2}$$

The ORR reaction rate is then defined as:

$$r(t) = k_{app}(t) \cdot \frac{c_1(t)}{1 + \frac{k_{app}(t)}{D/(\delta(t)/2)}} \qquad \text{A. 3}$$

B. Parameter values used in the cNFR model simulations

The parameter values are listed in Table B.1.

Table B.1 – Simulation values of the model parameters and constants

Parameter / Constant	Value
c_{bulk}	1.18 mol m^{-3}
c_{dl}	0.55 F m^{-1}
D	$1.9 \cdot 10^{-9}$ m^2 s^{-1}
k_{e1}	$4 \cdot 10^{-9}$ m s^{-1}
R_Ω	$1 \cdot 10^{-3}$ Ω m^2
α	0.5
ν	1.01·10-6 m^2 s^{-1}
E^θ	1.222 V
F	96485 C mol^{-1}

C. Additional Cost-Benefit indicator diagrams

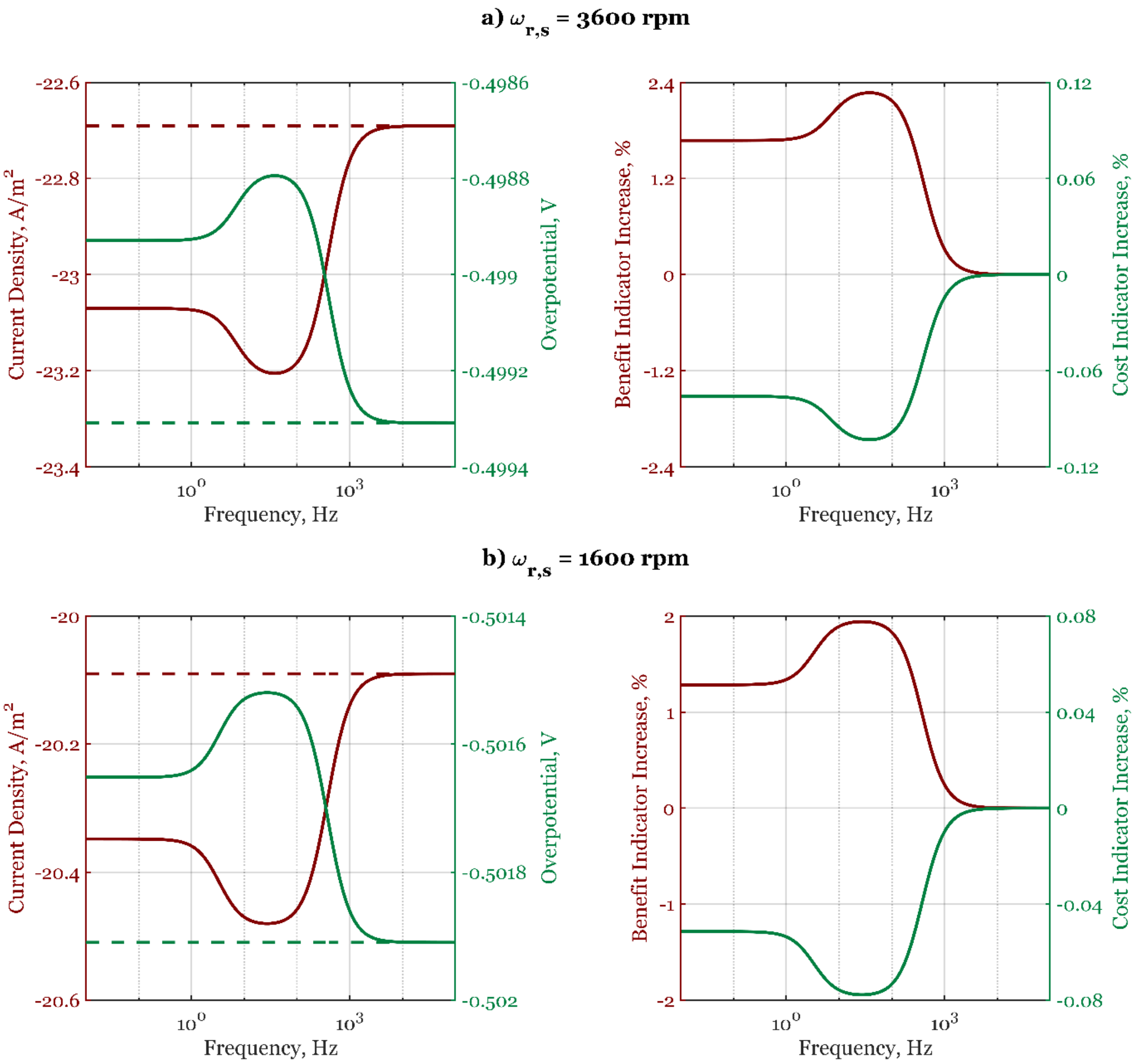

Figure C.1 - The mean current density and overpotential values (left side) for steady-state (dashed line) and dynamic regime (solid line), and the corresponding dynamic regime benefit and cost indicator increases relative to the steady-state regime (right side) when electrode potential is periodically changed with the amplitude of 5% around the value E_s = 0.6 V, and for steady-state electrode rotation rates: a) $\omega_{r,s}$ = 3600 rpm, and b) $\omega_{r,s}$ = 1600 rpm, in the frequency range of 10^{-2} to 10^5 Hz

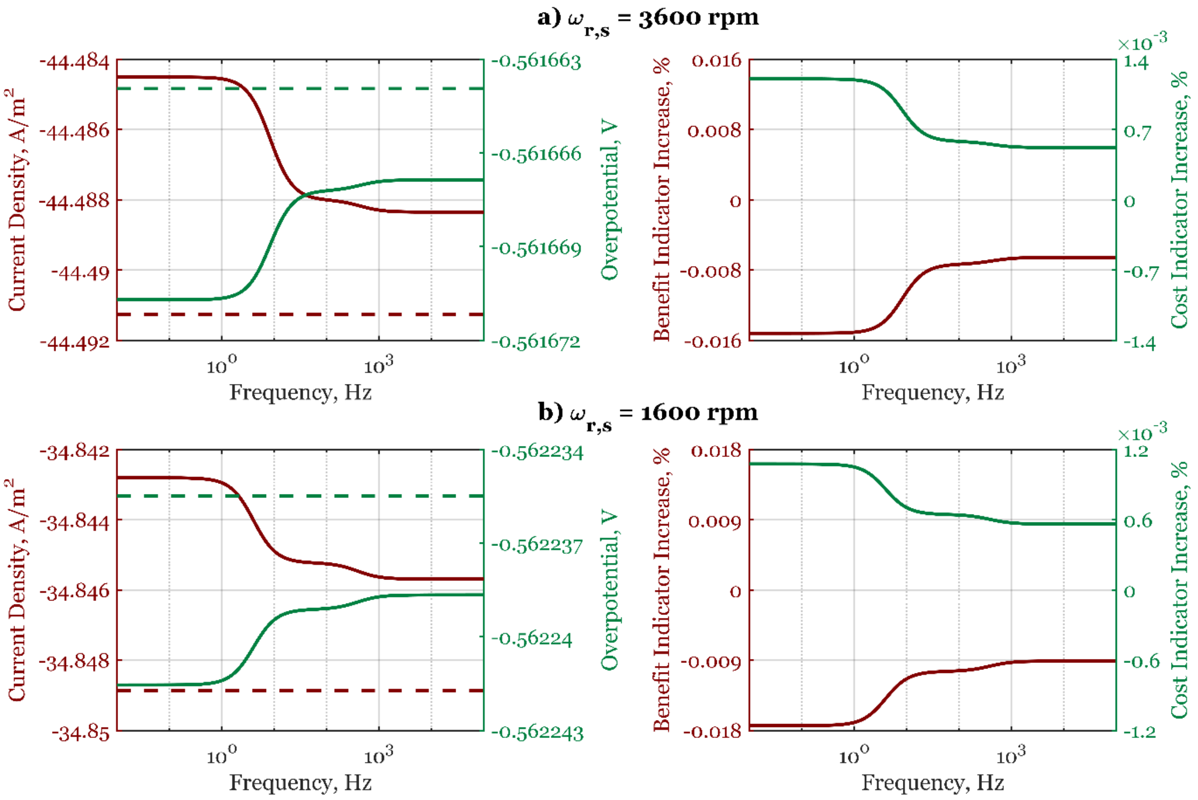

Figure C.2 - The mean current density and overpotential values (left side) for steady-state (dashed line) and dynamic regime (solid line), and the corresponding dynamic regime benefit and cost indicator increases relative to the steady-state regime (right side) when electrode rotation rate is periodically changed with the amplitude of 5%, steady-state electrode potential E_s = 0.6 V, and for steady-state electrode rotation rates: a) $\omega_{r,s}$ = 3600 rpm and b) $\omega_{r,s}$ = 1600 rpm, in the frequency range of 10^{-2} to 10^5 Hz
